# Correction: Visual orientation discrimination skills are tightly linked with specific aspects of human intelligence

**DOI:** 10.1371/journal.pone.0299165

**Published:** 2024-02-14

**Authors:** Kyriaki Mikellidou, Nefeli Lambrou, Ellada Georgiou, Marios Avraamides

There are errors in the Funding section. The correct Funding statement is: This project has received funding from and the Marie Skłodowska-Curie programme Grant agreement No 797603 (Peripheality; K.M., N.L., M.A) and from the European Union’s Horizon 2020 Research and Innovation Programme under Grant Agreement No 739578 and the Government of the Republic of Cyprus through the Deputy Ministry of Research, Innovation and Digital Policy (M.A.). The funders had no role in study design, data collection and analysis, decision to publish, or preparation of the manuscript.
