# Supplementary material for: Visual orientation discrimination skills are tightly linked with specific aspects of human intelligence
Source: PLoS One. 2023 Oct 17;18(10):e0289590. doi: 10.1371/journal.pone.0289590 (PMC10581472; doi:10.1371/journal.pone.0289590)
Supplement: S1 Appendix — (DOCX) [file pone.0289590.s001.docx]

**Appendix I**

******

***Supplementary Supplementary Figure 1***

*Two example psychometric functions from participants S39 and S91, yielding JNDs of 4.4 (point of subjective equality=45.2) and 2.9 degrees of visual angle (point of subjective equality=42.7), respectively.*

**Appendix II**

We report that the four subtests were positively and significantly correlated with one another (Supplementary Figure 2). The highest correlation was observed between the two verbal subtests, Similarities and Verbal Reasoning (Supplementary Figure 2B, r=0.573, p≤0.001). Block Design (Supplementary Figure 2D, r=0.406, p≤0.001) and Matrix Reasoning (Supplementary Figure 2F, r=0.397, p≤0.001) showed moderate correlations with Similarities. The weakest correlation was observed between individual normalised scores of the Verbal Reasoning and the Matrix Reasoning subtests (Supplementary Figure 2C, r=0.331, p≤0.001). This is in line with the fact that these two subtests turned out to be independent factors in our multiple linear regression model, explaining the variance with observed in JND. Scores in the two non-verbal subtests, Matrix Reasoning and Block Design were also moderately correlated (Supplementary Figure 2E, r=0.375, p≤0.001). Finally, scores in the Block Design were positively correlated with those of the Verbal Reasoning subtest (Supplementary Figure 2A, r=0.398, p≤0.001).

***Supplementary Figure 2***

*Individual normalised scores from the four subtests correlated against each other (N=92). Triangles indicate males, stars indicate females. All subtests are correlated with one another. A. Block design vs. Verbal Reasoning (r=0.398, p<0.001), B. Similarities vs. Verbal Reasoning (r=0.573, p<0.001). C. Matrix Reasoning vs. Verbal Reasoning r=0.331, p=0.001 D. Similarities vs. Block Design (r=0.406, p<0.001) E. Matrix Reasoning vs. Block Design (r=0.375, p<0.001), F. Matrix Reasoning vs. Similarities (r=0.397, p<0.001).*

**Appendix III**

All four subtests were found to be significantly and negatively correlated with JND. As shown in Supplementary Figure 3A, normalised Verbal Reasoning scores correlated negatively with JND (R=-0.31, p=0.002), showing that participants with an enriched vocabulary have better orientation discrimination skills (i.e., smaller JND values). There were also negative correlations between normalised Similarities scores and JND (Supplementary Figure 3C, R=-0.27, p=0.009) and Block Design with JND (Supplementary Figure 3B, R=-0.27, R^2^=0.1, p=0.008). Individual scores from the Matrix Reasoning subtest showed the strongest negative correlation of all subtests with JND (Supplementary Figure 3D, R=-0.32, R^2^=0.17, p=0.002).

***Supplementary Figure 3***

*Individual normalised scores from the four subtests are significantly correlated with JND (N=92). Verbal subtests illustrated in pink, non-verbal subtests in green. Triangles indicate males, stars indicate females. A. Verbal Reasoning normalised scores show a good negative correlation with orientation discrimination abilities (R^2^=0.15, p=0.006). B. Block Design normalised scores show a weaker correlation with orientation discrimination abilities (R^2^=0.1, p=0.025). C. Similarities normalised scores show a negative correlation with orientation discrimination abilities, similar to B (R^2^=0.1, p=0.032). D. Matrix Reasoning normalised scores show the strongest negative correlation with JND (R^2^=0.17, p=0.004).*
