# Supplementary material for: Visual orientation discrimination skills are tightly linked with specific aspects of human intelligence
Source: PLoS One. 2023 Oct 17;18(10):e0289590. doi: 10.1371/journal.pone.0289590 (PMC10581472; doi:10.1371/journal.pone.0289590)
Supplement: Attachment — Submitted filename: ResponsetoReviewers.pdf [file pone.0289590.s002.pdf]

We would like to thank both reviewers for their positive attitude and insightful feedback regarding our manuscript. We have made amendments to take care of all the issues raised and due to this we believe that the manuscript is now at a better shape than it was at the initial submission.

Kind Regards,

Dr. Kyriaki Mikellidou

Reviewer #1: Evaluation
In this paper Mikellidou et al. investigate whether a basic visual function, such as visual orientation discrimination, can be correlated with high-order cognitive abilities measured through the Intelligent Quotient (IQ). The authors found a negative correlation between these measures, specifically lower precision in visual orientation discrimination was associated with lower level in particular subtests of Verbal and Non-Verbal IQ. Finally they conclude that these association are not causative but they demonstrate the relationship between basic sensory processing that could in some way influence the develop of individual cognitive factors .

The study appears to have been well executed and the writing is clear and communicates the main points well. I really appreciated the clarity of the introduction. I think that some points should be addressed or clarified before the study is suitable for publication.

Methods

Visual Discrimination task : Is the position of the standard stimulus randomized across different trials, in order to avoid sequential order effects?

Yes, the standard stimulus was randomly presented either first or second to avoid sequential order effects and we now specify this in the text.

why the authors choose to present the stimuli in periphery rather than in fovea? This for my opinion would be a way to avoid eye movements of the subjects.

This is exactly the reason; we wanted to avoid excessive eye movements and we know clarify this in the text (page 4)

Results

in the first part of the results would be useful to have, together with SD of the different subtests , also the range of values, moreover I think that raw scores are not informative about the level of performance because they are not scaled for age. For that reason, I suggest to the authors to show scaled score rather than raw scores.

As suggested by the reviewer, we are now reporting scaled values and their range in the first part of the results.

Figure 2: I suggest to the authors to make Axis in figure 2 squares to have a better visualization of the correlation. Additionally In the figure 2 R stands for r-pearson ? if it is the case I suggest to use lowercase letter.

We have made the axis square for better visualisation and have also changed R to r, in order to indicate Pearson's r.

Looking in figure 2 is possible to identify subjects that showed a Verbal IQ below normality range (below scaled scores of 80), indeed this is reflected on the Total IQ scaled scores there are some subjects that showed an overall scores below 80. I therefore suggest that the authors consider eliminating these subjects because they have IQs at the lower end of the norm and could generate a bias in the results.

We understand why the reviewer is raising this point, and while we did notice a few participants with scores below normality when we checked for outliers using a standardised method (values that are 3 standard deviations above or below our sample mean, so anything between 66-131) all of our datapoints fell between this range. In fact, our lowest value is 74.5 and our highest is 122. In order to avoid any perceived "cherry-picking" from our side, we opted to leave the dataset as is.

Again for the analysis performed on the effect of degrees on IQ , the authors should use scaled scores rather than raw. Are there difference in visual orientation discrimination threshold across the different type of degrees?

We now use scaled scores to assess differences between degrees and also report that there are no differences in orientation discrimination threshold between the two types of degrees.

In order to give the reader a clearer and more comprehensive view of the results, I suggest the authors use a table containing the results of the multiple regression. Following this suggestion would be useful have an information of the amount of variance of Visual Orientation Threshold explained by the IQ subtests, so I would suggest to specify it in the presentation of the results.

We now present the results of multiple regression in a table and clarify that the amount of variance explained by the two significant IQ tests is 15%.

Reviewer #2: The authors of this interesting and well written article investigate the relationship between a simple visual discrimination task, which should be indicative of the functioning of low levels of vision, and the performance obtained on an intelligence test, in its verbal and non-verbal components. The article reveals a certain correlation between the two indicators and provides a broad discussion on what may be the effects of other factors not necessarily linked to the variables taken into consideration, such as working memory.

I think the work can be accepted after major revision, which I indicate below.

1) Visual discrimination task. Authors should better specify why they chose this specific test. The presentation time of the two gabors is quite long, and may allow the subjects to make eye movements: what reason, procedural or theoretical, led them to choose this time? Also, were the location and interval of the standard stimulus and test stimulus randomized on a trial-trial basis? Probably the authors could write a more accurate caption of figure 1 where to insert the various information.

We now explain why we use a long(ish) presentation time; this had to do with the fact that our sample comprised inexperienced participants which found it challenging to carry out the task with shorter presentation times. We also expanded the figure legend to enhance clarity.

2) The authors, before proceeding to describe the results, should insert a brief description of the statistical analysis carried out, specifying for example that they used the Pearson test which does not assume the normality of the data.

We now have a small subsection "Data analysis" which provides this information.

3) In any case, I would also report whether or not the data obtained are normally distributed.

We thank the reviewer for this comment and we now report that some of the variables are not normally distributed and this is why we use statistical tests that do not require the assumption of normality. For the comparison of the degrees we now report Mann-Whitney U results because of this (please note that no changes have been observed from our initial submission).

4) The correlations should be made, in my opinion, on the normalized data and not on the raw ones.

The correlations are now performed on the normalised data.

5) Figure 2 should show normalized data and the axes should be squared.

Figure 2 does show normalised data and the axes are now squared for better visualisation.

---

6. PLOS authors have the option to publish the peer review history of their article (what does this mean?). If published, this will include your full peer review and any attached files.
